# Usability of NewSTEPs Data for Assessing the Characteristics of Infants with Newborn Screening Disorders

**DOI:** 10.3390/ijns8030042

**Published:** 2022-07-19

**Authors:** Amel Omari, Sarah L. Reeves, Lisa A. Prosser, Melissa S. Creary, Ayesha Ahmad, Kao-Ping Chua

**Affiliations:** 1 Department of Health Behavior and Health Education, University of Michigan School of Public Health, Ann Arbor, MI 48109, USA; oamel@umich.edu; 2 Department of Pediatrics, Susan B. Meister Child Health Evaluation and Research Center, University of Michigan Medical School, Ann Arbor, MI 48109, USA; sleasure@med.umich.edu (S.L.R.); lisapros@med.umich.edu (L.A.P.); 3 Department of Epidemiology, University of Michigan School of Public Health, Ann Arbor, MI 48109, USA; 4 Department of Health Management and Policy, University of Michigan School of Public Health, Ann Arbor, MI 48109, USA; mcreary@umich.edu; 5 Department of Pediatrics, Division of Pediatric Genetics, Metabolism and Genomic Medicine, University of Michigan Medical School, Ann Arbor, MI 48109, USA; ayeshaah@med.umich.edu

**Keywords:** newborn screening, disorders, race and ethnicity, data repository, continuous data improvement

## Abstract

Most state newborn screening programs in the U.S. currently contribute case data to the Newborn Screening Technical Assistance and Evaluation Program (NewSTEPs). To assess the usability of these data for research, we examined the completeness of key variables, particularly race and ethnicity. Data included 24,129 cases of 34 newborn screening disorders from 45 states available in NewSTEPs as of 31 August 2020. Birth years of cases ranged between 2006 and 2020. Rates of missing data for sex, gestational age, birth weight, and race/ethnicity were 3.8%, 31.7%, 7.0%, and 39.7%, respectively. After excluding 21 states for which ≥50% of cases had missing data on race and/or ethnicity, 16,010 cases from 24 states remained. The disorders with the highest proportions in which cases were recorded as Hispanic ethnicity/any race were methylmalonic acidemia (48.7%) and maple syrup urine disease (45.7%). Analyses indicated that sex and birth weight data in NewSTEPs are reasonably complete, but missing data are common for gestational age and race/ethnicity. Despite this, our analyses revealed several novel associations between race/ethnicity and newborn screening disorders, such as the high burden of maple syrup urine disease among Hispanic patients. This demonstrates the potential usefulness of NewSTEPs for research if investments in higher-quality data are made.

## 1. Introduction

Each year, roughly 4 million U.S. newborns are screened for rare, life-threatening congenital disorders for which early detection and treatment are essential. In the U.S., responsibility for newborn screening is held by individual state newborn screening programs, which identify which conditions will be included in the newborn screening panel. Most states typically screen for most or all of the disorders in the Recommended Uniform Screening Panel (RUSP), a list of disorders that the Secretary of the U.S. Department Health and Human Services recommends states screen for in newborn screening programs. Some states screen for additional disorders beyond those included in the RUSP [1].

Historically, the decentralized, state-based nature of newborn screening resulted in data fragmentation, making it challenging to answer basic questions such as the incidence and demographic characteristics of infants with newborn screening disorders across states. For example, Feuchtbaum and colleagues [2] used data from a single state to examine racial and ethnic distributions within newborn screening disorder incidence. One of the goals of the Newborn Screening Technical Assistance and Evaluation Program (NewSTEPs), a program of the Association of Public Health Laboratories funded by the U.S. Health Services Research Administration starting in 2012, was to provide a central repository for newborn screening data across states [3]. 

Few peer-reviewed studies have analyzed the usability of NewSTEPs data for population-level research [4,5]. In this study, we examined the completeness of key demographic variables in NewSTEPs data. We particularly focused on the variable for the recorded race and ethnicity of infants with newborn screening disorders because high-quality data on race and ethnicity are crucial to understand the degree to which the differential incidence of newborn screening disorders may contribute to health inequities at birth. 

## 2. Materials and Methods

### 2.1. Study Sample

Our data included cases contributed by 45 of the 53 U.S. states, districts, and territories to NewSTEPs by 31 August 2020 (for brevity, we will refer to these geographic units simply as “states”). Each data point represents an infant with a confirmed diagnosis of one of 34 of the 35 disorders included in the RUSP as of July 2018; hearing loss is the single RUSP disorder not included. In 2020, the median number of disorders screened for among the 45 states was 32.5 (range: 30–34); 14 states screened for all 34 disorders.

The 45 states each signed memorandums of understanding to contribute data to NewSTEPs between 2014 and 2019 (median: 2015). However, some states only contribute data on cases that have occurred after memorandums were signed. Additionally, some states only contribute data on selected disorders, and the timeliness of data availability varies by state. Consequently, data only represent a subset of all cases detected by the 45 states by 31 August 2020.

Data elements include encrypted patient identifiers, disorder, birth year, sex, and clinical information, such as birth weight, gestational age, and family history. Data also include encrypted state, district, or territory identifiers and the regional genetic network to which the state belongs. Geographic origin of cases at a more granular level than region is encrypted to protect the privacy of patients with extremely rare disorders. Race is coded as White, Black or African American, Native American, Asian, and Native Hawaiian or Pacific Islander; multiple races can be coded. Ethnicity is coded separately as Hispanic/Latino/Spanish or not. In our analyses, we created seven categories for race and ethnicity: White alone/non-Hispanic, Black or African alone/non-Hispanic, Hispanic/any race, Asian alone/non-Hispanic, Native Hawaiian or Pacific Islander alone/non-Hispanic, Native American alone/non-Hispanic, and multiracial/non-Hispanic. According to NewSTEPs staff, race and ethnicity data refer to the mother in some states versus the infant in others. However, NewSTEPs does not compile information on the coding practices of each state.

### 2.2. Statistical Analysis

We used descriptive statistics to assess sample characteristics and to calculate the rate of missing data for sex, birth weight, and gestational age. We also calculated the proportion of cases with missing data for race, ethnicity, or both, overall and by disorder. To describe the racial and ethnic distribution of newborn screening disorders, we eliminated states for which ≥50% of cases across all disorders had missing race or ethnicity data, then calculated the proportion of the remaining cases in each of the seven racial and ethnic categories. This analysis balanced the need to maximize sample size and generalizability with the need to eliminate states contributing little information on race and ethnicity. We conducted analyses using RStudio version 4.1.3 (R Studio Team, Boston, MA, USA).

## 3. Results

### 3.1. Sample Characteristics

As of 31 August 2020, NewSTEPs data included 24,129 cases of the 34 RUSP disorders from 45 states. The state reporting the smallest number of total cases reported 16 cases. The state reporting the largest number of total cases reported 3066 cases. The median number of cases reported by states was 403. Birth years of cases ranged between 2006 and 2020, but most cases were born in 2015–2020.

Table 1 shows characteristics of the 24,129 cases, including percent missingness for variables with missing data. The regional genetics network accounting for the greatest proportion of cases (21.4%) was the New York-MidAtlantic Consortium for Genetics and Newborn Screening Services, which is comprised of New York and mid-Atlantic states such as Maryland, the District of Columbia, Puerto Rico, and the U.S. Virgin Islands. Rates of missing data for sex, gestational age and birth weight were 3.8%, 31.7%, and 7.0%, respectively. Of the 24,129 cases, 30.9% had missing data for race, 37.8% had missing data for ethnicity, and 39.7% had missing data for race and/or ethnicity. Among the 34 RUSP disorders, the median percentage of cases with missing data for race and/or ethnicity was 38.4% (25–75th percentile: 31.6–42.4%). 

### 3.2. Racial and Ethnic Distribution of Newborn Screening Disorders across All Reporting States

Table 2 displays the percentage of the 24,129 cases in each racial and ethnic category. Disorders varied in rarity. Looking across all 45 states, there were four cases of each of the rarest disorders in this dataset (3-hydroxy-3-methyglutaric aciduria and trifunctional protein deficiency) and 6979 cases of the most common disorder in this dataset (congenital hypothyroidism). The median number of cases in any of the 34 disorders, submitted to the data repository by any of the 45 states, was 112.

### 3.3. Racial and Ethnic Distribution of Newborn Screening Disorders among States with More Complete Data on Race and Ethnicity

Among the 45 states, the median percentage of cases with missing data for race and/or ethnicity was 44.1% (25th–75th percentile: 29.7–83.6%). To report the racial and ethnic distribution of cases from states with the most complete data, we excluded all cases from 21 states in which ≥50% of cases across all disorders had missing data for race and/or ethnicity. This resulted in the exclusion of 8119 cases (33.6%). The remaining 24 states contributed 16,010 cases. 

As shown in Table 3, 35.8% of the 16,010 cases were White/non-Hispanic, 23.8% were Black or African-American/Non-Hispanic, 16.6% were Hispanic/any race, and 18.1% of cases had missing race and/or ethnicity. The disorders with the highest proportion of cases recorded as Hispanic/any race were methylmalonic acidemia (48.7%) and maple syrup urine disease (45.7%). The disorders with the highest proportions of cases recorded as Black or African-American/non-Hispanic were presence of Hb S (71.0%) and mucopolysaccharidosis I (70.3%). 

## 4. Discussion

To our knowledge, this is the first analysis to assess the completeness of several key variables in NewSTEPs data. Findings suggest that data on sex and birth weight are reasonably complete, with rates of missing data of 3.8% and 7.0%, respectively, suggesting that NewSTEPs data can be used in research examining the distribution of these characteristics among infants with newborn screening disorders. However, data for gestational age were missing for 31.7% of cases, while data for race and/or ethnicity were missing for 37.8% of cases. This suggests that the utility of these data for research is more limited, particularly as the limited sample sizes and high rates of missingness for some disorders would pose challenges for commonly used statistical methods to account for missing data, such as multiple imputation. 

For race and ethnicity specifically, there were important limitations other than missing data. First, states were not identified owing to confidentiality restrictions. Consequently, the underlying number of newborns in each racial and ethnic category could not be calculated, precluding assessments of the incidence of newborn screening disorders among newborns in these categories. 

Second, race and ethnicity were not consistently measured among states. To address this limitation, state newborn screening programs should consider implementing standardized methods of data collection on race and ethnicity. Ideally, programs would record the self-reported race and ethnicity of both parents and the parent-described race and ethnicity of the infant. As information on maternal race and ethnicity is included in official reports of U.S. births [6], having the same information in NewSTEPs data could facilitate comparisons between the racial and ethnic distribution of all U.S. infants and those with newborn screening disorders. Importantly, implementing standardized data collection methods is costly and logistically challenging. Given limited state budgets, it may be challenging for state governments to invest the resources necessary to ensure newborn screening programs can either collect high-quality data or link to sources with high-quality data, such as vital records. At a minimum, however, states should provide detailed information on how they collect and report race and ethnicity data. 

Despite the limitations of race and ethnicity data in NewSTEPs, this study also highlights the potential usefulness of these data for research, particularly if improvements in data quality occur. For example, to our knowledge, this analysis is the first to suggest that mucopolysaccharidosis type I so disproportionately affects Black Americans when detected by newborn screening, or that maple syrup urine disease so disproportionately affects Hispanic Americans. While other datasets are potentially available for analyzing the racial and ethnic distribution of newborn screening disorders, including data from individual states [2], NewSTEPs is the only dataset of which we are aware that contains race and ethnicity data from infants with newborn screening disorders from the vast majority of U.S. states. A national database is particularly useful for studying the rarest RUSP disorders.

Importantly, we explicitly caution against using NewSTEPs data or the results from our analyses as evidence that race and ethnicity are genetic constructs. The false notion that race and ethnicity are determined by genetics, as opposed to social constructs, has been a hallmark of unethical “race science”, including eugenics [7,8,9]. Rather, the reported differences in this study may generate hypotheses for research into the etiologies of health inequities. For example, racial and ethnic disparities in the receipt of newborn screening and confirmation of disease may exist despite the near-universal nature of newborn screening. Future studies could explore whether such disparities drive the differences reported in this study. A more complete understanding of the racial/ethnic distribution of RUSP disorders could also facilitate an analysis of whether resources dedicated to treating and studying rare disorders are equitably distributed across burdened subpopulations. Such questions have been investigated with respect to cystic fibrosis and sickle cell anemia [10,11], but racial and ethnic disparities in research investment have not been evaluated across all RUSP disorders.

In summary, our analyses suggest that NewSTEPs data could represent a valuable database for research on the characteristics of infants with newborn screening disorders, particularly sex and birth weight. The utility of NewSTEPs data is more limited for gestational age and race and ethnicity owing in part to high rates of missing data. However, even with these limitations, we were able to demonstrate novel associations between race and ethnicity and certain newborn screening disorders, illustrating the substantial potential of NewSTEPs data for research. It is our hope that demonstrating these associations will motivate continued investments in the NewSTEPs repository, as well as improvements in data quality, so that this potential can be unlocked. It is also our hope that our analysis will motivate a broader assessment of the quality of data in newborn screening programs across the world.

## Figures and Tables

**Table 1:** Characteristics of infants in 45 states with newborn screening disorders in NewSTEPs data as of 31 August 2020 (*n* = 24,129 cases).

Characteristics	Number (%)
**Sex**	
Male	11,649 (48.3%)
Female	11,561 (47.9%)
Missing	919 (3.8%)
**Birth year**	
2006–2011	29 (0.1%)
2012–2014	5380 (22.3%)
2015–2017	10,763 (44.6%)
2018–2020	7957 (33.0%)
**Regional Collaborative**	
Heartland Genetics and Newborn Screening Collaborative	2600 (10.8%)
Mountain States Genetics Regional Collaborative	4784 (19.8%)
New England Genetics Collaborative	476 (2.0%)
New York-Mid-Atlantic Consortium for Genetics and Newborn Screening Services	5058 (21.0%)
Southeast NBS and Genetics Collaborative	4648 (19.3%)
The Region 4 Genetics Collaborative	4235 (17.6%)
Western States Genetic Services Collaborative	2328 (9.7%)
**Birth weight**	
0–1500 g	986 (4.1%)
1501–2499 g	2177 (9.0%)
2500–3499 g	13,010 (53.9%)
≥3500 g	6260 (25.9%)
Missing	1696 (7.0%)
**Gestational age**	
≤24 weeks	106 (0.4%)
25–31 weeks	592 (2.5%)
32–36 weeks	1811 (7.5%)
≥37 weeks	13,962 (57.9%)
Missing	7658 (31.7%)
**Race**	
White alone	9533 (39.5%)
Black alone	5767 (23.9%)
Asian alone	896 (3.7%)
Native American alone	121 (0.5%)
Pacific Islander or Native Hawaiian alone	37 (0.2%)
Multi-racial	318 (1.3%)
Missing	7457 (30.9%)
**Ethnicity**	
Hispanic, Latino, or Spanish	3162 (18.3%)
Not Hispanic, Latino, or Spanish	11,843 (68.4%)
Missing	9124 (37.8%)
**Race/ethnicity**	
White, non-Hispanic	6217 (25.8%)
Black, non-Hispanic	4141 (17.2%)
Hispanic, any race	3162 (13.1%)
Asian, non-Hispanic	729 (3.0%)
Pacific Islander or Native Hawaiian, non-Hispanic	18 (0.1%)
Native American, non-Hispanic	68 (0.3%)
Multi-racial, non-Hispanic	213 (0.9%)
Unknown race and/or unknown ethnicity	9581 (39.7%)

**Table 2:** Racial and ethnic distribution of patients with newborn screening disorders from 45 states (*n* = 24,129 cases).

Disorder	Total Cases	White, Non-Hispanic (*n*, %)	Black, Non-Hispanic (*n*, %)	Hispanic, Any Race (*n*, %)	Asian, Non-Hispanic (*n*, %)	Pacific Islander or Native Hawaiian, Non-Hispanic (*n*, %)	Native American, Non-Hispanic (*n*, %)	Multi-Racial, Non-Hispanic (*n*, %)	Unknown Race or Ethnicity (*n*, %)
3-Hydroxy-3-methyglutaric aciduria	ND	ND	ND	ND	ND	ND	ND	ND	ND
3-Methylcrotonyl-CoA carboxylase deficiency	272	75 (27.6)	25 (9.2)	54 (19.9)	11 (4.0)	ND	ND	ND	105 (38.6)
Argininosuccinic aciduria	73	28 (38.4)	ND	10 (13.7)	ND	ND	ND	ND	28 (38.4)
Beta-ketothiolase deficiency	11	ND	ND	ND	ND	ND	ND	ND	ND
Biotinidase deficiency	606	239 (39.4)	25 (4.1)	82 (13.5)	7 (1.2)	ND	ND	ND	251 (41.4)
Carnitine uptake/carnitine transport defect	99	33 (33.3)	7 (7.1)	12 (12.1)	7 (7.1)	ND	ND	ND	39 (39.4)
Citrullinemia, type I	111	29 (26.1)	10 (9.0)	23 (20.7)	11 (9.9)	ND	ND	ND	34 (30.6)
Classic galactosemia	541	167 (30.9)	28 (5.2)	66 (12.2)	10 (1.8)	ND	ND	ND	265 (49.0)
Classic phenylketonuria	859	391 (45.5)	9 (1.0)	87 (10.1)	8 (0.9)	ND	ND	7 (0.8)	357 (41.6)
Congenital adrenal hyperplasia	1162	312 (26.9)	55 (4.7)	355 (30.6)	35 (3.0)	ND	8 (0.7)	11 (0.9)	386 (33.2)
Congenital hypothyroidism	6976	1921 (27.5)	541 (7.8)	1144 (16.4)	334 (4.8)	11 (0.2)	34 (0.5)	56 (0.8)	2935 (42.1)
Critical congenital heart disease	755	283 (37.5)	110 (14.6)	81 (10.7)	34 (4.5)	ND	ND	7 (0.9)	238 (31.5)
Cystic fibrosis	4492	1826 (40.7)	135 (3.0)	709 (15.8)	40 (0.9)	ND	5 (0.1)	70 (1.6)	1706 (38.0)
Glutaric acidemia type I	144	44 (30.6)	11 (7.6)	26 (18.1)	7 (4.9)	ND	ND	ND	52 (36.1)
Hemoglobin—no structural variant ^a^	99	ND	ND	ND	38 (38.4)	ND	ND	ND	50 (50.5)
Holocarboxylase synthase deficiency	5	ND	ND	ND	ND	ND	ND	ND	ND
Homocystinuria	17	7 (41.2)	ND	ND	ND	ND	ND	ND	5 (29.4)
Isovaleric acidemia	113	30 (26.5)	13 (11.5)	19 (16.8)	5 (4.4)	ND	ND	ND	41 (36.3)
Long-chain L-3 hydroxyacyl-CoA dehydrogenase deficiency	39	14 (35.9)	ND	6 (15.4)	ND	ND	ND	ND	17 (43.6)
Maple syrup urine disease	88	18 (20.5)	6 (6.8)	33 (37.5)	ND	ND	ND	ND	27 (30.7)
Medium-chain acyl-CoA dehydrogenase deficiency	942	412 (43.7)	25 (2.7)	117 (12.4)	17 (1.8)	ND	ND	9 (1.0)	362 (38.4)
Methylmalonic acidemia (cobalamin disorders)	23	ND	ND	8 (34.8)	ND	ND	ND	ND	8 (34.8)
Methylmalonic acidemia (methylmalonyl-CoA mutase)	57	ND	ND	23 (40.4)	8 (14.0)	ND	ND	ND	18 (31.6)
Muco-polysaccharidosis I	133	18 (13.5)	90 (67.7)	9 (6.8)	ND	ND	ND	ND	14 (10.5)
Pompe	180	66 (36.7)	22 (12.2)	6 (3.3)	10 (5.6)	ND	ND	ND	72 (40.0)
Presence of Hb S ^b^	5010	53 (1.1)	2731 (54.5)	161 (3.2)	31 (0.6)	ND	5 (0.1)	17 (0.3)	2011 (40.1)
Presence of other Hb variant ^c^	515	10 (1.9)	206 (40.0)	9 (1.7)	80 (15.5)	ND	ND	9 (1.7)	198 (38.4)
Propionic acidemia	74	16 (21.6)	5 (6.8)	15 (20.3)	ND	ND	ND	ND	35 (47.3)
Severe combined immunodeficiencies	363	92 (25.3)	46 (12.7)	43 (11.8)	ND	ND	ND	ND	174 (47.9)
Spinal muscular atrophy	25	7 (28.0)	ND	ND	ND	ND	ND	ND	11 (44.0)
Trifunctional protein deficiency	ND	ND	ND	ND	ND	ND	ND	ND	ND
Tyrosinemia, type I	25	ND	ND	6 (24.0)	ND	ND	ND	ND	12 (48.0)
Very long-chain acyl-CoA dehydrogenase deficiency	250	83 (33.2)	18 (7.2)	32 (12.8)	8 (3.2)	ND	ND	ND	106 (42.4)
X-linked adrenoleukodystrophy	62	26 (41.9)	ND	10 (16.1)	6 (9.7)	ND	ND	ND	18 (29.0)
TOTAL	24,129	6217 (25.8)	4141 (17.2)	3162 (13.1)	729 (3.0)	18 (0.1)	68 (0.3)	213 (0.9)	9581 (39.7)

^a^ Alpha thalassemia major, Hb H, beta thalassemia major; ^b^ Hb S/B+ Th, Hb S/C, Hb SS, Hb S/B0Th, not known, S/other; ^c^ Hb C, Hb D, Hb E, Hb O-Arab, other Hb disease. ND – not displayed owing to cell sizes <5.

**Table 3:** Racial/ethnic distribution of patients with newborn screening disorders from 25 states (*n* = 16,010 cases).

Disorder	Total Cases	White, Non-Hispanic (*n*, %)	Black, Non-Hispanic (*n*, %)	Hispanic, Any Race (*n*, %)	Asian, Non-Hispanic (*n*, %)	Pacific Islander or Native Hawaiian, Non-Hispanic (*n*, %)	Native American, Non-Hispanic (*n*, %)	Multi-Racial, Non-Hispanic (*n*, %)	Unknown Race or Ethnicity (*n*, %)
3-Hydroxy-3-methyglutaric aciduria	ND	ND	ND	ND	ND	ND	ND	ND	ND
3-Methylcrotonyl-CoA carboxylase deficiency	191	69 (36.1)	24 (12.6)	45 (23.6)	8 (4.2)	ND	ND	ND	43 (22.5)
Argininosuccinic aciduria	52	28 (53.9)	ND	8 (15.4)	ND	ND	ND	ND	9 (17.31)
Beta-ketothiolase deficiency	10	ND	ND	ND	ND	ND	ND	ND	ND
Biotinidase deficiency	398	222 (55.8)	23 (5.8)	53 (13.3)	5 (1.3)	ND	ND	ND	93 (23.4)
Carnitine uptake/carnitine transport defect	72	29 (40.3)	7 (9.7)	9 (12.5)	6 (8.3)	ND	ND	ND	20 (27.8)
Citrullinemia, type I	81	28 (34.6)	8 (9.9)	20 (24.7)	10 (12.4)	ND	ND	ND	11 (13.6)
Classic galactosemia	513	338 (65.9)	9 (1.8)	66 (12.9)	8 (1.6)	ND	ND	6 (1.2)	86 (16.8)
Classic phenylketonuria	342	151 (44.2)	26 (7.6)	45 (13.2)	10 (2.9)	ND	ND	ND	105 (30.7)
Congenital adrenal hyperplasia	849	294 (34.6)	51 (6.0)	339 (39.9)	35 (4.1)	ND	8 (0.9)	10 (1.2)	112 (13.2)
Congenital hypothyroidism	4357	1794 (41.2)	510 (11.7)	902 (20.7)	284 (6.5)	8 (0.2)	25 (0.6)	48 (1.1)	786 (18.0)
Critical congenital heart disease	634	283 (44.6)	110 (17.4)	79 (12.5)	34 (5.4)	ND	ND	7 (1.1)	119 (18.8)
Cystic fibrosis	2874	1652 (57.5)	125 (4.4)	629 (21.9)	40 (1.4)	ND	ND	62 (2.2)	363 (12.6)
Glutaric acidemia type I	102	41 (40.2)	8 (7.8)	24 (23.5)	6 (5.9)	ND	ND	ND	21 (20.6)
Hemoglobin—no structural variant ^a^	60	ND	ND	ND	30 (50.0)	ND	ND	ND	19 (31.7)
Holocarboxylase synthase deficiency	5	ND	ND	ND	ND	ND	ND	ND	ND
Homocystinuria	14	7 (50.0)	ND	ND	ND	ND	ND	ND	ND
Isovaleric acidemia	80	26 (32.5)	12 (15.0)	17 (21.3)	5 (6.3)	ND	ND	ND	17 (21.3)
Long-chain L-3 hydroxyacyl-CoA dehydrogenase deficiency	24	13 (54.2)	ND	ND	ND	ND	ND	ND	ND
Maple syrup urine disease	70	17 (24.3)	6 (8.6)	32 (45.7)	ND	ND	ND	ND	11 (15.7)
Medium-chain acyl-CoA dehydrogenase deficiency	617	369 (59.8)	22 (3.6)	101 (16.4)	16 (2.6)	ND	ND	7 (1.1)	102 (16.5)
Methylmalonic acidemia (cobalamin disorders)	10	ND	ND	ND	ND	ND	ND	ND	ND
Methylmalonic acidemia (methylmalonyl-CoA mutase)	39	ND	ND	19 (48.7)	8 (20.5)	ND	ND	ND	6 (15.4)
Mucopolysaccharidosis I	128	18 (14.1)	90 (70.3)	9 (7.0)	ND	ND	ND	ND	9 (7.0)
Pompe	122	65 (53.3)	20 (16.4)	ND	9 (7.4)	ND	ND	ND	20 (16.4)
Presence of Hb S ^b^	3498	53 (1.5)	2485 (71.0)	138 (4.0)	30 (0.9)	ND	5 (0.1)	14 (0.4)	772 (22.1)
Presence of other Hb variant ^c^	358	7 (2.0)	190 (53.1)	8 (2.2)	67 (18.7)	ND	ND	7 (2.0)	76 (21.2)
Propionic acidemia	43	15 (34.9)	ND	15 (34.9)	ND	ND	ND	ND	6 (14.0)
Severe combined immunodeficiencies	225	89 (39.6)	45 (20.0)	36 (16.0)	ND	ND	ND	ND	47 (20.9)
Spinal muscular atrophy	13	7 (53.9)	ND	ND	ND	ND	ND	ND	ND
Trifunctional protein deficiency	ND	ND	ND	ND	ND	ND	ND	ND	ND
Tyrosinemia, type I	17	ND	ND	6 (35.3)	ND	ND	ND	ND	ND
Very long-chain acyl-CoA dehydrogenase deficiency	ND	ND	ND	ND	ND	ND	ND	ND	ND
X-linked adrenoleukodystrophy	191	69 (36.1)	24 (12.6)	45 (23.6)	8 (4.2)	ND	ND	ND	43 (22.5)
TOTAL	52	28 (53.9)	ND	8 (15.4)	ND	ND	ND	ND	9 (17.31)

^a^ Alpha thalassemia major, Hb H, beta thalassemia major; ^b^ Hb S/B+ Th, Hb S/C, Hb SS, Hb S/B0Th, not known, S/other; ^c^ Hb C, Hb D, Hb E, Hb O-Arab, other Hb disease. ND – not displayed owing to cell sizes <5.

## Data Availability

Restrictions apply to the availability of these data. Data were obtained from NewSTEPs at the Association of Public Health Laboratories and may be requested through NewSTEPs’ published request process (https://www.newsteps.org/data-repository/data-requests).
